# Dentists' knowledge of dental trauma based on the International Association of Dental Traumatology guidelines: An Australian survey

**DOI:** 10.1111/edt.12761

**Published:** 2022-05-23

**Authors:** Nilesh Madhukant Jadav, Paul V. Abbott

**Affiliations:** ^1^ UWA Dental School The University of Western Australia Perth WA Australia

**Keywords:** avulsion, crown‐root fracture, dental trauma, lateral luxation, root fracture, treatment

## Abstract

**Background/Aim:**

Traumatic dental injuries are the result of impact injuries to the teeth and/or soft and hard tissues within and around the vicinity of the oral cavity and pose a very serious public health dilemma. The aim of this study was to appraise the level of knowledge of dentists in Australia regarding the management of traumatic dental injuries based on the International Association of Dental Traumatology (IADT) guidelines.

**Methodology:**

A link to an electronic questionnaire investigating personal and professional information and twelve questions about dental trauma was distributed by email using the Qualtrics Survey Software to ensure anonymity of the respondents, to members of the Australian Dental Association. The respondents were grouped according to demographic characteristics and practice profiles. Data were evaluated by the Student’s *T* test or one‐way ANOVA with post hoc testing using Fisher's least significant difference, with the *α* level set at 5%.

**Results:**

A total of 180 complete responses were obtained. The overall mean number of correct answers was 7.55 ± 1.91 from a maximum possible score of twelve. Gender, year of primary dental qualification*,* dentist identity (general dentist or specialist), area of main practice or region worked by the practitioner did not significantly affect the mean scores. However, increased knowledge of the IADT guidelines was significantly associated with the number of trauma cases treated and the dentists' self‐reported knowledge.

**Conclusions:**

The overall knowledge of Australian dentists regarding the management of traumatic dental injuries based on the IADT guidelines was generally good but it was also deficient in some areas.

## 1 INTRODUCTION

Traumatic dental injuries (TDI's) are the result of an impact to the teeth and/or soft and hard tissues within and around the vicinity of the oral cavity.^1^ The most frequent injury is an enamel fracture whilst the most commonly affected tooth is the maxillary central incisor.^2^ Some of the more common causes of these injuries include road traffic accidents,^3^ collisions and falls associated with leisure or sporting activities^4^ and abuse that may be physical or violent in its nature.^5^

Traumatic dental injuries pose a very serious public health dilemma, and indications are that the prevalence of these injuries may well exceed dental caries and periodontal disease as the single most significant health issue affecting the young in the future.^6^ Epidemiological data have indicated a high incidence of traumatic dental injuries to both the primary and permanent dentitions. Approximately one third of pre‐school children have experienced trauma to their deciduous dentition whilst a quarter of school children and almost one third of all adults have a history of trauma to their permanent dentition.^7^, ^8^

The initial management and subsequent maintenance of traumatized teeth requires adequate knowledge of the injury, exceptional clinical skills and appropriate long‐term follow‐up.^6^ Failure to address the injury in a prompt and appropriate manner could have devastating and far reaching consequences—such as pain, functional and aesthetic problems as well as psychological issues affecting not only the patient but also the parents.^9^ The role of the dental practitioner is therefore pivotal and crucial in affecting the long‐term outcome of treatment and potentially the patient's quality of life.^10^

The International Association of Dental Traumatology (IADT) has published a series of four treatment guidelines, based on expert group discussions and literature reviews to aid health professionals in providing the optimal management of TDI's. The first publication in the current guidelines provides a general introduction,^11^ the second paper looks at managing fractures and luxations of permanent teeth,^12^ the third covers avulsion of permanent teeth^13^ and the fourth article focuses on the management of traumatic injuries to the primary dentition.^14^

Although this information is readily available, numerous international studies and several systematic reviews have been published in the literature highlighting insufficient levels of knowledge possessed by dentists regarding the management of TDI's.^6^, ^15^, ^16^, ^17^, ^18^, ^19^, ^20^, ^21^, ^22^, ^23^, ^24^, ^25^, ^26^ These studies evaluating TDI knowledge are essential as the data gleaned may be used to formulate health system policies and to develop strategies to improve dental education.^2^ Therefore, the aim of this study was to appraise the level of knowledge of dentists in Australia regarding the management of TDI's based on the IADT guidelines.

The null hypothesis was that there was no difference in levels of knowledge of dental trauma among Australian dentists based on the International Association of Dental Tramatology guidelines.

## 2 MATERIALS AND METHODS

This research was approved by the University of Western Australia's Human Research Ethics Committee in accordance with the requirements of the National Statement on Ethical Conduct in Human Research (National Statement) and the policies and procedures of the University of Western Australia (RA/4/20/6238).

A sample size calculation based on approximately 24,000 registered dentists in Australia, a 95% confidence interval and a 10% margin of error indicated that a minimum of 96 responses were required. A link to the electronic questionnaire, distributed using the Qualtrics Survey Software (Provo, UT, USA; Version 03.2020) to ensure anonymity of the respondents, was sent by email to members of the Australian Dental Association. The survey commenced in June 2020 and was open for responses for a period of three months. Participation in the study was voluntary.

The questions were based on the 12 questions used by Hartmann et al.,^2^ for a survey of dentists in Brazil. The same questions were used in order to allow direct comparisons of the dentists' responses and knowledge levels. The questionnaire was considered to be valid as it had been tested and used by Hartmann et al.^2^ However, it was examined by several endodontic and paediatric dental specialists and tested on general dentists in order to assess items for clarity of wording as the language was changed from Portuguese to English. Based on their responses, the wording of some questions were modified for clarity. The final questionnaire (Supplementary File S1) was divided into two parts: Part I was used to identify the socio‐demographic and professional profiles of respondents ‐ age, gender, years of experience, highest level of education, area of practice, main practice setting, number of dental trauma cases previously treated, and their self‐reported knowledge of dental trauma (SKDT). Participants were asked to rate their knowledge as ‘very good’, ‘good’, ‘acceptable’ or ‘low’. Part II comprised 12 questions related to dento‐alveolar trauma according to the 2020 IADT guidelines, first published at the end of May 2020.^11^, ^12^, ^13^, ^14^

The level of knowledge was assessed using a scoring system that assigned one point for each correct answer and zero points for incorrect answers for the 12 questions in Part II of the survey. All participants received a final score with a maximum possible score of 12 points. These scores were categorized as either Low (0–3), Acceptable (4–6), Good (7–9) or Very Good (10–12).

The results were initially analysed by descriptive statistics, with analyses carried out using Microsoft® Excel® 2016 MSO (16.0.4549.1000) 64‐bit. Statistical testing was carried out using IBM SPSS STATISTICS, version 25 software (IBM Corp,). The Student’s T test was used to analyse dichotomous variables (Specialist v GDP, city v regional, and gender), and one‐way ANOVA with post hoc testing using Fisher's least significant difference (LSD) was applied for groups of more than two possible answers (year graduated, area of practice, number of cases treated in the last 12 months, and self‐reported knowledge). Testing for association between the self‐reported knowledge and the actual score obtained from the survey questions, which was considered to be the main outcome of the study, was assessed by the Spearman's Rank Correlation Coefficient test. The α level was set at 5%.

## 3 RESULTS

A total of 180 complete responses were obtained. The mean age of respondents was 42.95 ± 13.38 years (range 24–75 years). The overall mean number of correct answers was 7.55 ± 1.91, indicating a good level of knowledge. The demographic data from the completed questionnaires and their associated mean scores are summarized in Table 1. Statistical analysis revealed no significant difference in mean scores between gender, year of primary dental qualification*,* dentist identity (general dentist or specialist), area of main practice or region worked by the practitioner.

**TABLE 1:** Demographic characteristics of respondents (*n* = 180)

Participants' responses	Number (%)	Mean score ± SD	*p*‐value
Gender			
Male	92 (51.1)	7.71 ± 1.74	.365
Female	82 (45.6)	7.45 ± 1.97	
Year of Primary Dental Qualification			
1950–59	1 (0.6)		.744
1960–69	1 (0.6)		
1970–79	13 (7.2)	8.23 ± 1.59	
1980–89	26 (14.4)	7.69 ± 1.59	
1990–99	30 (16.7)	7.57 ± 2.08	
2000–2009	47 (26.1)	7.26 ± 1.62	
2010–2019	62 (34.4)	7.74 ± 1.95	
General Dentist v Specialist			
General dentist	149 (82.8)	7.53 ± 1.92	.762
Specialist	31 (17.2)	7.65 ± 1.89	
Main Area of Practice			
Academic	10 (5.6)	7.70 ± 1.83	.993
Private	128 (71.1)	7.69 ± 1.72	
Public	26 (14.4)	7.73 ± 1.69	
Retired	2 (1.1)	4.50 ± 6.36	
Not specified (excluded)	14 (7.8)	–	
Region of Country			
City	133 (73.9)	7.57 ± 1.90	.801
Regional	47 (26.1)	7.48 ± 1.97	
Trauma Cases Treated in Previous 12 Months			
None	24 (13.3)	6.83 ± 1.15	.035
1	17 (9.4)	7.53 ± 1.62	
2–4	62 (34.4)	7.47 ± 2.04	
5–9	26 (14.4)	7.42 ± 2.04	
10+	38 (21.1)	8.29 ± 1.47	
Not specified (excluded)	13 (7.2)	–	
Self‐reported Knowledge			
Low	15 (8.3)	7.20 ± 1.93	.089
Acceptable	92 (51.1)	7.41 ± 1.87	
Good	50 (27.8)	7.54 ± 1.47	
Very Good	21 (11.7)	8.52 ± 2.64	
Not specified (excluded)	2 (1.1)	–	

Almost all (94.4%) of the respondents had clinical experience in dealing with dental trauma within the previous 12 months. Approximately one‐fifth (21.1%) of the respondents had treated ten or more cases within the previous 12 months. These practitioners had significantly higher mean scores than respondents who had treated no trauma cases (*p* = .002) and those who had treated between two and four cases (*p* = .026) within the previous 12 months. However, no significant difference existed in the groups of participants who had treated one case (*p* = .145) and five to nine cases (*p* = .057).

When participants were questioned about their self‐reported knowledge, slightly more than a quarter (27.8%) of the respondents self‐rated their knowledge as ‘good’ and 11.7% rated their knowledge as ‘very good’. Correlation coefficient testing revealed a low to moderate, but significant, association between their mean scores and each of the self‐reported knowledge groups (ρ = 0.167, *p* = .026). Although ANOVA testing did not show that self‐reported knowledge was reflective of the actual knowledge (*p* = .089), further post hoc testing did show that those who considered their knowledge to be ‘very good’ had significantly higher mean knowledge scores than those who had self‐rated their knowledge as ‘good’ (*p* = .046), ‘acceptable’ (*p* = .016) and ‘low’ (*p* = .039).

The most recent IADT guidelines were used to formulate the main subject of each question, as well as the correct answers regarding the management of each injury or scenario. Table 2 lists the number of correct answers for each question based on these guidelines. Colour coding was used, based on the traffic light system, which is a modified version of the KAP‐Heat Map devised by Tewari et al.^26^ to highlight the different levels of awareness. Question 6 (mid‐third root fracture) and Question 8 (subluxation with no response to pulp sensibility testing) had the highest number of correct answers (85.0% and 85.6%, respectively), while Question 4 (avulsed tooth with complete root development) and Question 12 (laterally luxated permanent tooth with alveolar bone fracture) had the lowest numbers of correct answers (27.8% and 35.6% respectively).

**TABLE 2:** Number of correct scores from the 180 participants for the 12 questions about the management of dental trauma based on the IADT guidelines using a colour‐coded traffic light system

Question	Case Scenario	No. of Correct Answers (%)
1	Avulsion	**151 (83.9)**
2	Storage solution for an Avulsed Tooth	**84 (46.7)**
3	Avulsion Open Apex	**126 (70.0)**
4	Avulsion Closed Apex	**50 (27.8)**
5	Avulsion without Bone Fracture	**104 (57.8)**
6	Root Fracture	**153 (85.0)**
7	Intrusion	**70 (38.9)**
8	Subluxation	**154 (85.6)**
9	Enamel, Enamel/Dentine Fracture, Subluxation, Concussion	**113 (62.8)**
10	Crown‐Root Fracture	**148 (82.2)**
11	Enamel/Dentine/Pulp Fracture, Closed Apex	**142 (78.9)**
12	Lateral Luxation, Bone Fracture	**64 (35.6)**

**≤ 25%**	**26‐50%**	**51‐75%**	**≥76%**

## 4 DISCUSSION

Treatment guidelines for dental trauma assist dental professionals in providing evidence based care in the most efficient manner. The correct application of this knowledge is therefore pivotal immediately after a traumatic dental injury to provide the best possible short‐ and long‐term outcomes.^11^ The aim of this study was to evaluate the knowledge of the management of dental trauma amongst Australian dentists based on the latest IADT guidelines that were published in 2020.^11^, ^12^, ^13^, ^14^

The participants' answers regarding the management of 12 dental trauma scenarios were used as the basis for assessing their knowledge of dental trauma management. The results indicated a non‐significant correlation between the actual knowledge and the year of primary dental qualification, with older dentists achieving higher mean scores. In contrast, studies from Brazil, the United Kingdom and the United Arab Emirates have reported an inverse relationship with younger practitioners possessing higher levels of knowledge.^2^, ^27^, ^28^, ^29^ An interesting finding from the current study was that recent graduates (that is, those who had graduated within the last 10 years) did not follow this trend which was similar to other studies that reported marginally better knowledge scores amongst more recent graduates.^2^, ^17^, ^19^, ^30^ A possible explanation for this could be related to the recent revisions of undergraduate training curricula with an increased focus on dental traumatology.^30^ A Polish study has also suggested that younger dentists are more likely to revise material in preparation for examinations relating to qualification for specialization.^31^

In accordance with previous studies, specialization did not contribute to a significant improvement in knowledge scores compared with general dentists.^29^, ^32^ However, Kostopoulou and Duggal^6^ found that specialization and additional training positively influenced knowledge about emergency treatment. Similarly, Hartmann et al.^2^ reported that dentists who held higher academic degrees (Masters and/or PhD) achieved significantly better results. Alyasi et al.^27^ and Hu et al.^18^ also found similar statistically significant results, but those studies only looked at the individual specialities of paediatric dentistry and endodontics respectively.

There were no significant differences in the mean scores between dentists working in rural areas and those working in metropolitan city areas. This is in accordance with Lund et al.^29^ although a comparable study from China reported better knowledge amongst dentists located in urban areas.^23^ In the current study, city dentists had marginally higher mean scores than dentists located in rural areas. This is somewhat surprising given that rural dentists, particularly in Australia, can be located hundreds, and in some instances, thousands of kilometres away from metropolitan areas, and as a result they are forced to treat dental trauma cases, whereas those more centrally based have the option to refer to hospitals or specialists, and therefore their exposure to treating TDI's would be expected to be lower.

As reported in previous studies, a proportional relationship was noted between the mean knowledge scores of participants and the number of cases previously treated.^2^, ^31^, ^33^ This study showed that dentists who had treated more than 10 cases in the preceding year had significantly higher mean scores than those who had treated fewer cases. This is likely to be a result of the greater clinical experience improving the dentists' skills.^2^

An important finding was that dentists who self‐evaluated their knowledge as being ‘very good’ had some of the highest mean knowledge scores. This is in agreement with Hartmann et al.^2^ and Zaleckiene et al.^30^ who both showed that higher levels of competence in treating dental trauma were associated with higher self‐reported knowledge. On the contrary, studies by Krastl et al.^19^ and Skaare et al.^34^ reported that self‐assessment was not reflective of actual knowledge. The results from the current study indicated a proportional increase in scores as the dentist's self‐rated knowledge increased. Although initial statistical analysis revealed no significant difference, further post hoc testing showed that dentists who perceived their knowledge to be ‘very good’ scored significantly higher than those who considered their knowledge as ‘good’, ‘acceptable’ or ‘low’. This finding may be explained by the low numbers of participants in the ‘low’ and ‘very good’ groups, which may have affected the statistical analysis.

In this study, 83.9% of the respondents correctly answered that immediate replantation of an avulsed tooth should ideally occur at the accident site which is in accordance with current IADT guidelines and indicates good knowledge pertaining to this type of injury.^13^ This is higher than in the studies reported by Cohenca et al.^16^ and Zhao and Gong^23^ who showed that 53.3% and 70.7%, respectively, of the respondents to their surveys would not replant the tooth in every case.

When asked about their preference for storing avulsed teeth if replantation cannot occur, approximately half (46.7%) of the respondents in the current study considered milk as the favoured storage medium. This was lower than previous studies which reported answers ranging from 60%–99%.^6^, ^18^, ^28^, ^32^ The second most common response (37.2% of respondents) to this question was to place the tooth in saliva. Saliva contains harmful enzymes and bacteria, which over time may damage the periodontal ligament cells. It is therefore recommended that saliva should only be used for short extra alveolar storage periods, with the saliva and tooth placed in a container or wrapped in plastic.^36^

The best time to start endodontic treatment for a tooth with a closed apex proved to be a controversial question in this study. Almost two thirds (61.7%) of the participants indicated that they would initiate root canal treatment within 7–10 days after replanting the tooth. This is similar to the findings of Krastl et al.^19^, and it was recommended in earlier versions of the IADT guidelines.^37^, ^38^ Those recommendations also included the use of calcium hydroxide as an intracanal medicament to prevent external inflammatory resorption. However, immediate or early placement of calcium hydroxide induces cell necrosis of the reparative cells as well as the resorbing cells. As a result, ankylosis and replacement resorption becomes the typical healing response.^39^ The last two versions of the IADT guidelines^13^, ^40^ include the above recommendation but they also included an alternative option which is to commence root canal treatment immediately after replanting and stabilising the avulsed tooth, followed by placement of a corticosteroid‐antibiotic intracanal medicament, if available. Bryson et al.^41^ showed that the use of a commercially available corticosteroid‐antibiotic paste known as Ledermix Paste (Haupt Pharma GmbH,) decreased the amount of resorption and resulted in greater areas with favorable healing compared with the immediate use of calcium hydroxide. This favorable response is due to the powerful anti‐inflammatory properties of the corticosteroid (triamcinolone) component and anti‐bacterial effects of the antibiotic (demeclocycline) component. As a result, its use should be considered as a first line medicament in the treatment of replanted avulsed teeth.^41^ It is important to recognize that Ledermix Paste is not commercially accessible worldwide, and as a result, the option to consider calcium hydroxide, although not recommended as the primary treatment in Australia, is available.

Almost two‐thirds (57.8%) of the participants would splint an avulsed tooth for approximately 2 weeks, which is an accordance with the current IADT guidelines.^13^ This was higher than previous studies, where figures ranged from 10% to 53%.^23^, ^32^, ^35^, ^42^ Just over one‐third (39.4%) of the respondents suggested splinting times of 6 weeks which was similar to the findings reported by Baginska and Wilczynska‐Borawska,^31^ but, unfortunately, this is a potential risk factor for ankylosis and replacement resorption.^43^

The prognosis for transverse fractures in the middle third of the root is good provided prompt treatment with close adaptation of the root fragments is instituted, with studies reporting an 80% healing rate if correctly performed.^44^ In the current study, 85.0% of the participants would only commence root canal treatment if there were clinical or radiographic signs of pulp necrosis and infection, which is in accordance with current IADT guidelines.^12^ In contrast, a previous Australian study reported that 45% of respondents would always provide root canal treatment for horizontal root fractures.^42^

Only 38.9% of the respondents would institute root canal treatment for an intruded tooth with a completely formed root, which is a cause for concern. This type of injury causes severe crushing of the neurovascular bundle, and pulp revascularization is unlikely to occur when the root is fully formed. As a result, there is a significant risk of pulp necrosis and infection,^42^ together with ankylosis and replacement resorption which may ultimately lead to eventual loss of the tooth.^2^

Following subluxation, 85.6% of the participants would refrain from initiating root canal treatment if the tooth did not respond to pulp sensibility testing which follows the IADT guidelines.^12^

Pulp capping or pulpotomy would be performed by 78.9% of the respondents following a complicated crown fracture. This is similar to the findings of Yeng and Parashos^42^ who reported that 86% of their participants would provide conservative pulp treatment (38% partial pulpotomy, 48% pulp capping).

Lateral luxation injuries damage both the periodontal ligament and the pulp. Therefore precise repositioning of the tooth is crucial in order to optimize periodontal ligament healing and possible pulp revascularization.^42^ In this study, only 35.6% of the participants would rigidly splint a laterally luxated tooth for 30 days. The current IADT guidelines^11^, ^12^ recommend a passive *flexible* splint using composite resin and stainless steel wire up to 0.4 mm in diameter for a period of 4 weeks, but then suggest that if there is also a fracture of the marginal bone or alveolar socket wall, additional splinting may be required.^12^ This is confusing for a number of reasons as, by definition, lateral luxation injuries usually also have bone fractures. The guidelines do not specify what is meant by ‘additional splinting’. Furthermore, Kwan et al.^45^ found that when the wire used for a composite/wire splint was greater than 0.4 mm in diameter, the splint was deemed to be a rigid splint. They concluded that a wire thickness of 0.4 mm was the clinical threshold between flexible and rigid splinting. Despite this finding, confusion still exists as to what is a flexible splint and what is a rigid splint. Ideally, this requires further research and clarification.

This study had several limitations. Firstly, there was a low response rate. Although data from different cities and specialties were included, the low response rate may not be representative of the dental profession in Australia as a whole, possibly leading to skewed results.^33^ It is also important to note that data, particularly from other countries or studies, should be interpreted with caution. Methodological differences together with possible inappropriate interpretation of the guidelines may make direct comparisons challenging. Lastly, the manner in which some of the questions were worded may have affected the response to certain questions, possibly leading to questionnaire bias.^42^

Evaluating dental trauma knowledge is critical, as data may be used to introduce health system policies and to implement new or improved educational strategies.^33^ It is therefore imperative that the principles of qualitative research are used to develop well‐designed primary studies, incorporating uniform study methods, assessments and reporting protocols. The heterogeneity in these studies can therefore be reduced, allowing the results to be viewed without caution.^26^

## 5 CONCLUSIONS

The knowledge of Australian dentists regarding the management of dental trauma injuries based on the IADT guidelines is generally good but it is also deficient in some areas. Knowledge levels were not statistically significantly associated with gender, year of graduation, main area of practice and region worked. However, dental trauma knowledge was significantly associated with the number of trauma cases treated and the dentists' self‐reported knowledge.

## AUTHOR CONTRIBUTIONS

NMJ and PVA contributed equally to the development of the research protocol and questionnaire. NMJ conducted the survey. Both NMJ and PVA analysed the results. NMJ drafted the first version of the paper. Both NMJ and PVA edited and finalised the article.

## CONFLICT OF INTEREST

Both authors declare that they have no conflicts of interest with this work.

## Supporting information


Appendix S1

## Data Availability

The data that support the findings of this study are available from the corresponding author upon reasonable request.
