# Supplementary material for: Dentists' knowledge of dental trauma based on the International Association of Dental Traumatology guidelines: An Australian survey
Source: Dent Traumatol. 2022 May 23;38(5):374–80. doi: 10.1111/edt.12761 (PMC9545508; doi:10.1111/edt.12761)
Supplement: Supplementary file 1 — Appendix S1 [file EDT-38-374-s001.docx]

**SUPPLEMENTARY FILE**

**Dentist’s knowledge of dental trauma based on the International Association of Dental Traumatology guidelines: An Australian survey**

This Supplementary File shows the questions and choices of answers used in the survey.

For the case scenarios in Questions 10-21, the correct answers, according to
the IADT guidelines (2020), are highlighted in red.

**1 – Gender:** Male, Female or Prefer not to disclose

**2 – Age:** Participants had to manually enter their response.

**3 – Where did you complete your primary dental training?** Australia, New Zealand, United Kingdom, North America, South America,
 Europe, Africa, Asia, Oceania (excluding Australia and New Zealand)

**4 – What year did you complete your primary dental qualification?** 1950-1959, 1960-1969, 1970-1979, 1980-1989, 1990-1999, 2000-2009,
 2010-2019

**5 – Are you a registered dental specialist?** Yes, No – if “Yes” selected, proceed to Questions 5 a, b, c

**5a - What type of registered specialist are you?** Dento-Maxillofacial Radiology, Endodontics, Oral and Maxillofacial Surgery,
 Oral Medicine, Oral Pathology, Oral Surgery, Orthodontics, Paediatric
 Dentistry, Periodontics, Prosthodontics, Public Health Dentistry (Community
 Dentistry), Special Needs Dentistry, Forensic Odontology

**5b - Where did you complete your specialist training?** Australia, New Zealand, United Kingdom, North America, South America,
 Europe, Africa, Asia, Oceania (excluding Australia and New Zealand)

**5c - What year did you complete your specialist training?** 1950-1959, 1960-1969, 1970-1979, 1980-1989, 1990-1999, 2000-2009,
 2010-2019

**6 – What is your main area of practice?** Private, Public, Academic, Retired

**7 – Where do you work?** Major Cities of Australia, Inner Regional Australia, Outer Regional Australia,
 Inner Remote Australia, Outer Remote Australia.

**8 – Have you ever treated any cases of dental trauma?** Yes, No – If “Yes” selected, proceed to Question 8a

**8a - If so, approximately how many in the last 12 months?** Participants had to manually enter their response.

**9 - How would you rate your knowledge about dental trauma?** Low, Acceptable, Good, Very Good

**10 – The best option for the emergency management of an avulsed tooth is:**

- Immediate replantation at the accident site
- Replantation in the dental office
- Replantation by a specialist
- I don't know how

**11 – What is the best solution in which to transport an avulsed tooth if it cannot be replanted?**

- Sterile gauze
- Tap water
- Hydrogen peroxide
- Milk
- Saline solution
- Alcoholic solution
- Patient's saliva
- Contact lens solution
- I don't know

**12 - The best time to start endodontic treatment for a tooth with incomplete root formation that was avulsed and replanted is when clinical and radiographic evidence of pulp necrosis and infection is observed.**

- True
- False
- I don’t know

**13 - The best time to start endodontic treatment for a tooth with complete root development that was avulsed and replanted, within 1 hour, is 7 to 10 days after replantation.**

- True
- False
- I don’t know

**14 – How long should the splint remain, on an avulsed tooth without an associated bone fracture?**

- 2 weeks
- 6 weeks
- I don’t know

**15 - In the case of a root fracture in the middle third of the root, how should you proceed?**

- Start endodontic treatment immediately
- Follow up and review the tooth with pulp sensibility tests and radiographs and only intervene endodontically if the tooth shows clinical and radiographic evidence of pulp necrosis and infection
- I don’t know

**16 - When an intrusion of a tooth with complete root formation occurs, there is a potential risk of tooth loss due to external resorption. Endodontic treatment is indicated in all cases because pulp revascularization will not occur.**

- True
- False
- I don’t know

**17 ­- In cases of subluxation in which pulp sensibility testing is negative, endodontic treatment should be started immediately:**

- True
- False
- I don’t know

**18 - In less serious traumatic incidents (enamel fractures, enamel and dentine fractures, subluxations, concussions), the time for follow-up visits are 6 to 8 weeks and 1 year after the trauma.**

- True
- False
- I don’t know

**19 - The most important factor to consider in the case of a crown/root fracture, in addition to the existence or not of pulp exposure, is the extension of the fracture line in an apical direction.**

- True
- False
- I don’t know

**20 - In a case of a crown fracture involving enamel and dentine with exposure of the pulp in a tooth with complete root development, what is the best treatment?**

- Endodontic treatment as soon as possible, avoiding total contamination of the dental pulp.
- Direct composite restoration with an adhesive system applied directly on the pulp.
- Direct pulp capping with Calcium Hydroxide or Mineral Trioxide Aggregate (MTA) and then restore the tooth.
- Evaluate the signs and symptoms of the patient and the macroscopic aspects of the pulp (consistency, colour and bleeding)- then, based on these and, if possible, perform direct pulp capping, or pulpotomy, using Calcium Hydroxide or MTA, and restore the tooth
- I don’t know

**21 - In a case of lateral luxation of a permanent tooth with alveolar bone fracture, what would be the ideal type and time for splinting?**

- Rigid splint for 7 days
- Rigid splint for 14 days
- Rigid splint for 30 days
- Flexible splint for 7 days
- Flexible splint for 14 days
- Flexible splint for 30 days
- I don’t know
